# Impaired Mitophagy Contributes to Pyroptosis in Sarcopenic Obesity Zebrafish Skeletal Muscle

**DOI:** 10.3390/nu17101711

**Published:** 2025-05-18

**Authors:** Xiangbin Tang, Yunyi Zou, Siyuan Yang, Zhanglin Chen, Zuoqiong Zhou, Xiyang Peng, Changfa Tang

**Affiliations:** Key Laboratory of Physical Fitness and Exercise Rehabilitation of Hunan Province, College of Physical Education, Hunan Normal University, Changsha 410012, China; txb_133@hunnu.edu.cn (X.T.); 2023101510304@hunnu.edu.cn (Y.Z.); yangsiyuan@hunnu.edu.cn (S.Y.); zhanglinchen@hunnu.edu.cn (Z.C.); zhouzuoqiong@hunnu.edu.cn (Z.Z.)

**Keywords:** sarcopenic obesity, mitophagy, pyroptosis, zebrafish

## Abstract

**Background:** Growing evidence suggests that the prevalence of sarcopenic obesity (SOB) is on the rise across the globe. However, the key molecular mechanisms behind this disease have not been clarified. **Methods:** In this experiment, we fed zebrafish a high-fat diet (HFD) for 16 weeks to induce sarcopenic obesity. **Results:** After a dietary trial, HFD zebrafish exhibited an obese phenotype with skeletal muscle atrophy and decreased swimming capacity. We demonstrated that mitochondrial content and function were abnormal in SOB zebrafish skeletal muscle. These results may be associated with the impairment of mitophagy regulated by the PTEN-induced putative kinase 1 (PINK1)/Parkin (PRKN) pathway. In addition, we also found that NOD-like receptor protein 3 (NLRP3)/gasdermin D (GSDMD) signaling was activated with the upregulation of NLRP3, GSDMD-NT, and mature-IL1β, which indicated that pyroptosis was induced in SOB zebrafish skeletal muscle. **Conclusions:** Our study identified that impaired mitophagy and pyroptosis were associated with the pathogenesis of SOB. These results could potentially offer novel therapeutic objectives for the treatment of sarcopenic obesity.

## 1. Introduction

The epidemic of obesity is still one of the major threats to global public health [1]. Obesity is linked to metabolic abnormalities, including hypertension, fatty liver diseases, and type 2 diabetes mellitus [2]. Obesity also contributes to metabolic disturbance, pro-inflammatory cytokine production, and insulin resistance in muscle tissue [3]. Prolonged consumption of an HFD can induce muscle atrophy with myofibrillar protein degradation, eventually leading to sarcopenic obesity [4,5,6]. Sarcopenic obesity (SOB), in combination with the deterioration of skeletal muscle function, can lead to decreased exercise capacity with an associated risk of disability, increased risks of cardiovascular disease, and overall mortality [7,8,9]. Epidemiological studies reveal that the prevalence of SOB among elderly populations aged 60+ has reached 10–30%. With the accelerating global population aging and the escalating obesity pandemic, SOB demonstrates not only a persistent surge in incidence rates but also emerging characteristics of earlier age of onset and broader population demographics. Projections indicate the global affected population may reach 100 to 200 million by 2050 [10], establishing this condition as an urgent public health challenge demanding immediate intervention [11]. Elucidating the pathogenic mechanisms and formulating prevention strategies for SOB holds profound practical significance in refining chronic disease management frameworks, optimizing healthcare resource allocation, and alleviating societal economic burdens.

Recently, an inflammatory necrosis was found by researchers and was named pyroptosis [12]. Different from other cell death modes, pyroptosis has its own characteristics in terms of morphology, occurrence, and regulatory mechanism [12,13]. The signaling molecules involved in pyroptosis include parts of the caspase family proteins (1, 4, 5, 11), gasdermin family proteins, and inflammasomes. The release of pro-inflammatory factors is an indispensable process during pyroptosis [14]. It is manifested as cell swelling until cell membrane rupture, resulting in the release of cell contents and then activating a strong inflammatory response [13,14]. Pyroptosis widely participates in a variety of disease development, such as infectious diseases [15], nervous system-related diseases [16], atherosclerosis [17], and cancer [18]. Recent research found that pyroptosis is also implicated in the pathogenetic mechanisms of sarcopenia [19]. The upregulated expression of pyroptosis factors (such as NLRP3, caspase-1, cle-caspase-1, and GSDMD-N) was found in the skeletal muscle of sarcopenic mice [20,21].

Recent studies have found that damaged mitochondria release ROS and mtDNA into the cytoplasm and then induce NLRP3 inflammasome activation, which subsequently leads to caspase-1-dependent pyroptosis [22,23]. Mitophagy is an evolutionarily conserved cellular process that removes dysfunctional or redundant mitochondria and plays a key role in maintaining mitochondrial homeostasis and cell survival [24]. Mitophagy could remove damaged mitochondria, thereby inhibiting the activation of pyroptosis [25,26]. The prevailing view is that there is a negative feedback regulatory mechanism between mitophagy and pyroptosis [27,28]. In sarcopenic obesity, dysregulated mitophagy was found in skeletal muscle [29]. However, whether mitophagy and pyroptosis mechanisms mediate the pathological development of SOB remains unclear.

Previous studies have demonstrated that a high-fat diet is an effective way to induce sarcopenic obesity in animal models [30,31,32,33]. Considering the high degree of conservation of skeletal muscle biology between humans and zebrafish [34,35], in this experiment, we induced SOB in zebrafish through 16 weeks of a high-fat diet. We explored the impacts of SOB on muscle quality and motor ability of zebrafish. We aimed to investigate whether mitophagy and pyroptosis mediated the pathological development of sarcopenic obesity.

## 2. Materials and Methods

### 2.1. Animal Culture Conditions and Experimental Design

The zebrafish used in this study were obtained from the China Zebrafish Resource Center. AB strain zebrafish (2 months old) were used in this experiment. All zebrafish were bred under light for 14 h at 28 °C in standard husbandry conditions.

Zebrafish were adaptively fed a standard diet (TP1FM21051; Trophic Animal Feed High-Tech Co., Ltd., Nantong, China) with 6% lipid content for 7 days prior to the start of the experiment. Following acclimatization, 60 size-matched zebrafish were selected and randomly allocated to two diet groups: a normal diet (6% fat; ND, *n* = 30) or an HFD (24% fat; HFD, *n* = 30). Zebrafish were fed three times per day. After 16 weeks, the following morphometric parameters were systematically recorded: body weight and body length. For swimming capacity assessment, both dietary group zebrafish were randomly selected. After 24 h of fasting, zebrafish were randomly anesthetized with 20 mg/L tricaine methanesulphonate and then sampled. Zebrafish tail muscles were stripped for the following experiments.

All experiments were performed in accordance with the Chinese guidelines for animal welfare and experimental protocols. Approval was obtained from the Animal Experiment Administration Committee of Hunan Normal University (Hunan, China; approval number 2018/046).

### 2.2. Micro-CT

The measurement method of body fat volume was described previously [36]. In brief, zebrafish were subjected to anesthesia with MS-222 and subsequently sandwiched between two wet sponges. The Micro-CT device (μCT-50; Scanco medical, Bassersdorf, Swizerland) was used for whole-body scans of zebrafish with a resolution of 14 μm. The three-dimensional (3D) representations of the adipose tissue were acquired through a 3D reconstruction with VG Studio Max (v2.1). Each group was implemented on four zebrafish.

### 2.3. Biochemical Analyses

For detecting triglyceride and total cholesterol, three zebrafish skeletal muscles (150 mg) from each diet were mixed and then homogenized in 0.9% normal saline (1:9, *v*/*v*) and centrifuged at 2000 rpm for 15 min. The supernatants were collected, and further tests were conducted. Biochemical parameters were measured using specific commercial kits (triglyceride assay kit, #A110-1-1; total cholesterol assay kit, #A111-1-1; Jiancheng Biotech Co., Nanjing, China).

### 2.4. Swimming Capacity and Oxygen Consumption Measurement

Zebrafish exercise capacity and oxygen consumption were conducted on a technical swimming tunnel respirator (Loligo Systems, Viborg, Denmark). During the test, the speed and oxygen consumption of zebrafish at each stage will be recorded and then calculated based on the following existing formula: *U*crit = *U*f + *U*S × (*T*f/*T*S), where *U*f (cm/s) is the highest velocity, *U*S (2.7 BL/s) is the velocity increasement, *T*f (min) is the time at the maximum swimming velocity before exhaustion, and *T*S (14 min) is the duration of each velocity increment. Maximal oxygen consumption (MO_2_) was calculated using AutoResp 1 software (Loligo Systems, Viborg, Denmark). More detailed information can be found in our previous study [37].

### 2.5. Open Field Test

During the field experiment, the animals were placed in small Petri dishes with a diameter of 8 cm, allowing them to swim freely. The swimming time of the animals was recorded for 3 min (15 min after entering the arena). After the optimization and implementation of the Loligotrack system, the swimming behavior within one minute was analyzed. The average swimming speed, average acceleration, and total distance of the zebrafish in 1 min were recorded.

### 2.6. Histological Analysis

Three zebrafish were selected for each group, and their skeletal muscle samples were preserved in a 4% paraformaldehyde solution for a duration of 24 h. Subsequently, these samples were embedded in paraffin and divided into 4 µm thick slices for H&E staining. The cross-sectional area (CSA) of muscle fibers was quantified using ImageJ software (version 1.54g).

Three skeletal muscle samples were preserved in 4% paraformaldehyde for a duration of 4 h and embedded in oct. Cryosections (8 µm thick) were fabricated on a cryostat and were then stabilized with 4% paraformaldehyde for an additional 30 min. The slides underwent a washing process in distilled water and were subsequently stained with Oil Red O for a duration of 15 min. Oil Red O staining was used to determine lipid content in skeletal muscle tissue (the red positive markers indicate lipids).

The visual data were obtained through a microscopic lens (Leica, Heidelberg, Germany) and subsequently subjected to analysis via ImageJ (NIH, Bethesda, MD, USA).

### 2.7. Transmission Electron Microscopy

Skeletal muscle tissue was fixed in 2.5% glutaraldehyde for 6–12 h and then manipulated according to the procedures described previously. The mass of mitochondria as well as the structure of muscle fibers were observed by transmission electron microscopy [30].

### 2.8. Western Blot

Zebrafish skeletal muscle tissues were subjected to high milling and subsequent centrifugation using a RIPA buffer, resulting in the isolation of protein-rich supernatant. The BCA test was employed to ascertain protein concentration. After electrophoresis, membrane transfer, and antibody incubation, the proteins were visualized utilizing a gel documentation apparatus (Tanon, Shanghai, China). The antibodies used were as follows: rabbit anti-GAPDH antibody (1:2000; Proteintech, Wuhan, China), rabbit anti-Atrogin1 antibody (1:1000; Abcam, Cambridge, UK), rabbit anti-muscle RING-finger protein-1 (MuRF1) antibody (1:1000; Abcam), rabbit anti-PINK1 (1:1500; Proteintech, Rosemont, IL, USA), rabbit anti-PRKN antibody (1:1000; Bioss, Beijing, China), rabbit anti-NLRP3 antibody (1:1500; Wanleibio, Shenyang, China), rabbit anti-GSDMD antibody (1:1500; Proteintech), and rabbit anti-IL-1β antibody (1:1500; Proteintech). The level of protein expression was adjusted to match the levels of GAPDH.

### 2.9. Mitochondrial Respiratory Function

Mitochondrial respiratory function was assessed using the Oroboros O2K high-resolution respirometry system (Oroboros Instruments GmbH, Innsbruck, Austria), following a standardized protocol adapted from published methodologies. Briefly, caudal skeletal muscle tissue (4 mg) was homogenized on ice in an MIR05 preservation buffer. The homogenate was transferred to the respirometry chamber, and baseline oxygen consumption was stabilized prior to sequential substrate additions. The following substrates and inhibitors were titrated into the system to evaluate specific electron transport chain (ETC) complexes: Complex I-linked respiration: 2 mM pyruvate, 2 mM malate, and 10 mM glutamate; ATP synthase activity: 2.5 mM ADP; Complex I inhibition: 1 μM rotenone; Complex II-driven respiration: 10 mM succinate; cytochrome c integrity test: 20 μM cytochrome c; Complex II inhibition: 5 mM malonic acid; Complex III inhibition: 2.5 μM antimycin A; and Complex IV activity: 0.8 mM ascorbate and 0.2 mM TMPD (artificial electron donors).

Real-time oxygen flux data were recorded using DatLab 7.4 software (Oroboros Instruments, Innsbruck, Austria) and normalized to tissue mass for quantitative analysis of ETC functional states.

### 2.10. Statistical Analysis

Data analysis was conducted utilizing GraphPad Prism software (version 9.0; San Diego, CA, USA). Data were expressed as means ± standard deviations. Unpaired *t*-tests were used to compare the mean values of the two groups (high-fat diet compared with the normal diet group). All experiments were repeated three times. Statistical significance was set at *p* < 0.05.

## 3. Results

### 3.1. Sixteen Weeks of a High-Fat Diet Induced Obesity in Zebrafish

**Figure 1:**
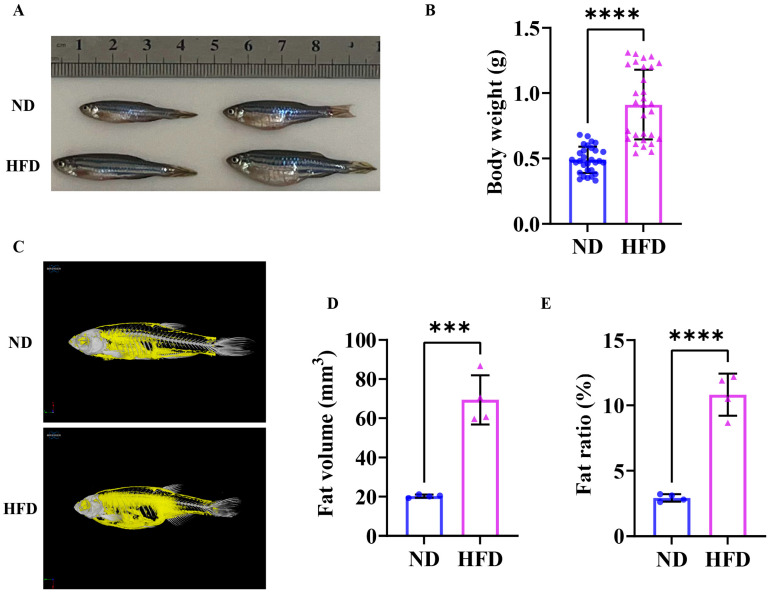
Zebrafish exhibited an obesity phenotype after 16 weeks of a high-fat diet. (**A**) Zebrafish morphology. (**B**) Body weight. (**C**) Micro-CT; the yellow shaded areas are fat (*n* = 4). (**D**) Fat volume (*n* = 4). (**E**) Fat ratio (*n* = 4). ***, *p* < 0.001, ****, *p* < 0.0001. ND, normal diet; HFD, high-fat diet.

The morphological change in zebrafish following a 16-week high-fat diet is shown in Figure 1A. The HFD enhanced the growth performance of fish due to its high energy density, potentially leading to increased body length. And, the body weight of zebrafish in the HFD group exhibited a significant increase when compared to the ND group (Figure 1B). The results of the Micro-CT of zebrafish in both groups are shown in Figure 1C; notable rises in both visceral and subcutaneous fat levels can be found in the HFD group. The fat volume (Figure 1D) and the fat ratio (Figure 1E) of HFD zebrafish were significantly higher than ND zebrafish. The findings revealed that 16 weeks of a high-fat diet induced obesity in zebrafish.

### 3.2. Sixteen Weeks of a High-Fat Diet Increased Lipid Content in Zebrafish Skeletal Muscle

**Figure 2:**
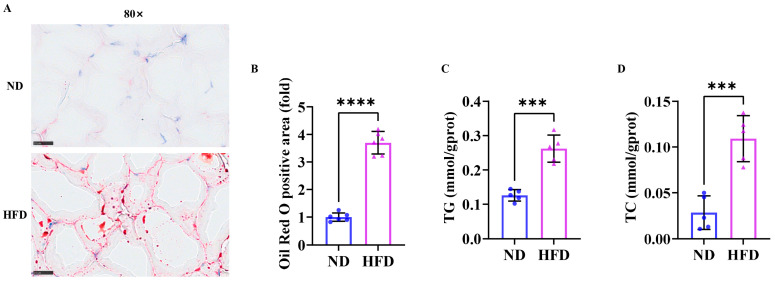
Fat accumulation in zebrafish skeletal muscle after 16 weeks of a high-fat diet. (**A**) Skeletal muscle Oil Red O staining (80×) (*n* = 3). (**B**) Quantitative results of skeletal muscle Oil Red O staining. (**C**) Triglycerides. (**D**) Total cholesterol. Scale bar, 25 μm. ***, *p* < 0.001, ****, *p* < 0.0001. ND, normal diet; HFD, high-fat diet.

The results of skeletal muscle Oil Red O staining (Figure 2A) showed that the lipid content (positive area, red) of zebrafish in the HFD group was significantly higher than that in the ND group (Figure 2B). Triglycerides (Figure 2C) and total cholesterol (Figure 2D) in the HFD group were also upregulated compared to the ND group. These results suggested that 16 weeks of a high-fat diet facilitated fat accumulation in obese zebrafish skeletal muscle.

### 3.3. Exercise Capacity Is Impaired in Obese Zebrafish

**Figure 3:**
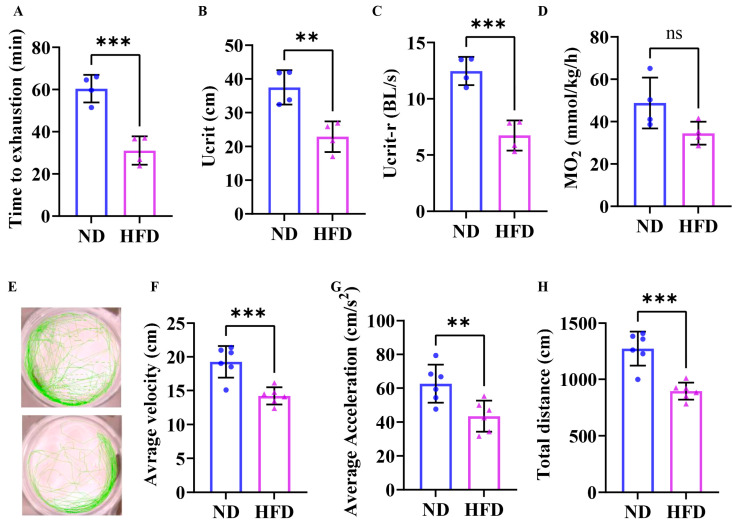
Impaired swimming capacity was found in obese zebrafish. (**A**) Exhaustion time (*n* = 4). (**B**) Critical swimming speed (*n* = 4). (**C**) Relative critical swimming speed (*n* = 4). (**D**) Maximum oxygen consumption (*n* = 4). (**E**) Motion trail of zebrafish in the open field test (*n* = 6). (**F**) Average speed. (**G**) Average acceleration. (**H**) Swimming distance. **, *p* < 0.01, ***, *p* < 0.001, ns, not significant. ND, normal diet; HFD, high-fat diet.

Critical swimming speed (Ucrit) is a key indicator of a fish’s sustained swimming ability, reflecting its aerobic metabolic capacity and muscle function. The results of the Ucrit test showed that the exhaustion time (Figure 3A), critical swimming speed (Figure 3B), and relative critical swimming speed (Figure 3C) of the HFD group were downregulated compared to the ND group, and the maximum oxygen consumption (Figure 3D) showed a tendency to decrease but did not reach statistical significance. As shown in 3E, the average speed (Figure 3F), average acceleration (Figure 3G), and swimming distance (Figure 3H) observed in the HFD group during the open field test were notably reduced compared to those exhibited by the ND group. The findings revealed that the exercise capacity was impaired in obese zebrafish.

### 3.4. Sixteen Weeks of a High-Fat Diet Induced Skeletal Muscle Atrophy in Zebrafish

**Figure 4:**
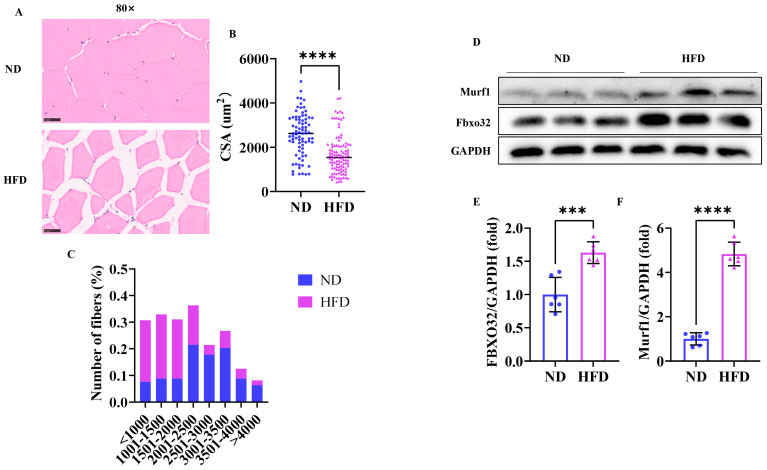
Skeletal muscle atrophy was found in obese zebrafish. (**A**) Skeletal muscle HE staining (80×). (**B**) Cross-sectional area of skeletal muscle fiber. (**C**) Skeletal muscle fiber size distribution map. (**D**) Western blot of Murf1 and Fbxo32 (*n* = 6). (**E**) Protein expression of FBXO32. (**F**) Protein expression of Murf1. Scale bar, 25 μm. ***, *p* < 0.001, ****, *p* < 0.0001. ND, normal diet; HFD, high-fat diet.

As shown in Figure 4A, HE staining displayed that the cross-sectional area of skeletal muscle fiber (Figure 4B) in HFD zebrafish was notably smaller compared to those in the ND group, and the cross-sectional area of skeletal muscle fiber in HFD zebrafish had a larger proportion in a smaller distribution range (Figure 4C). The Western blot results showed (Figure 4D) that the expression levels of skeletal muscle atrophy markers Murf1 and Fbxo32 in the HFD group were markedly increased (Figure 4E,F). Collectively, these results demonstrated that 16 weeks could induce muscle atrophy and sarcopenic obesity in zebrafish.

### 3.5. Sixteen Weeks of a High-Fat Diet Induced Impaired Skeletal Muscle Mitochondrial Function in Obese Zebrafish

**Figure 5:**
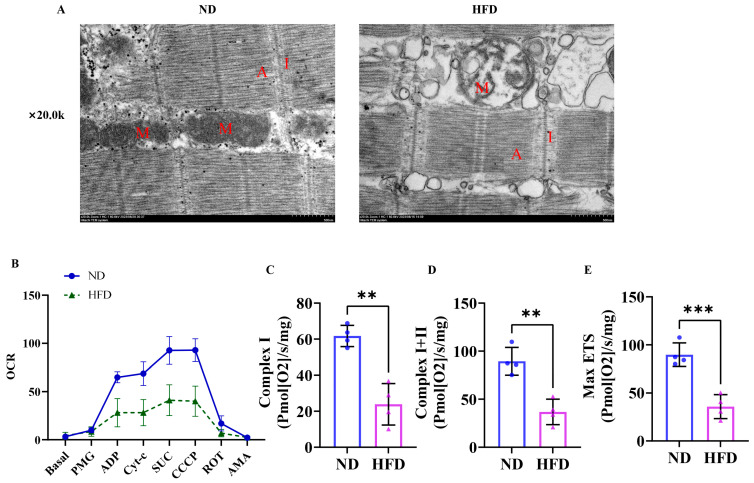
Skeletal muscle mitochondrial dysfunction was found in obese zebrafish. (**A**) Transmission electron microscopy (TEM) of skeletal muscle. (**B**) Mitochondrial respiration instrument test (*n* = 4). (**C**) Complex I. (**D**) Complex I + II. (**E**) MAXETS. **, *p* < 0.01, ***, *p* < 0.001. M, mitochondria; A, anisotropic; I, isotropic; ND, normal diet; HFD, high-fat diet.

We assessed mitochondrial damage by analyzing skeletal muscle mitochondrial morphology in high-fat diet-induced obese zebrafish. As depicted in Figure 5A, mitochondria in the HFD group displayed severe swelling and cristae loss compared to the ND group. The results of the mitochondrial respiration function test are shown in Figure 5B. The HFD zebrafish skeletal muscle mitochondrial respiratory Complex I (Figure 5C), I + II (Figure 5D) and the maximum electron transport capacity (Figure 5E) were significantly lower than those of the ND group. These findings suggested that mitochondrial morphology was impaired and mitochondrial function was inhibited in SOB zebrafish.

### 3.6. Sixteen Weeks of a High-Fat Diet Induced Impaired Skeletal Muscle Mitophagy in Obese Zebrafish

**Figure 6:**
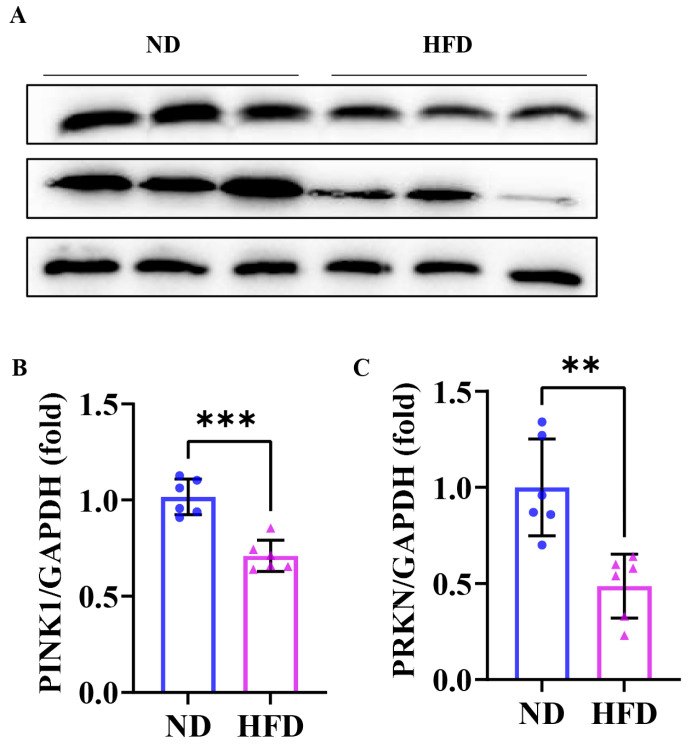
Impaired skeletal muscle mitophagy was found in obese zebrafish. (**A**) Western blot of PINK1 and PRKN (*n* = 6). (**B**) Protein expression of PINK1. (**C**) Protein expression of PRKN. **, *p* < 0.01, ***, *p* < 0.001. ND, normal diet; HFD, high-fat diet.

The Western blot results showed (Figure 6A) that the expression levels of the mitophagy proteins PINK1 and PRKN in the skeletal muscle of zebrafish in the HFD group were significantly increased compared with those in the ND group (Figure 6B,C). These results indicated that damaged mitophagy was found in SOB zebrafish.

### 3.7. Sixteen Weeks of a High-Fat Diet Induces Skeletal Muscle Pyroptosis in Obese Zebrafish

**Figure 7:**
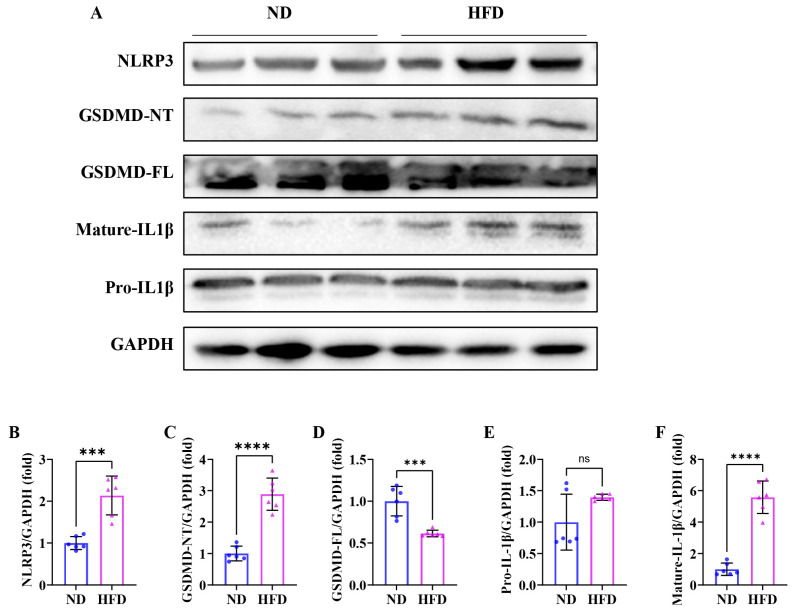
Pyroptosis occurred in obese zebrafish skeletal muscle. (**A**) Western blot of NLRP3, GSDMD-NT, GSDMD-FL, mature-IL1β, and pro-IL1β (*n* = 6). (**B**) Protein expression of NLRP3. (**C**) Protein expression of GSDMD-NT. (**D**) Protein expression of GSDMD-FL. (**E**) protein expression of pro-IL1β and (**F**) Protein expression of mature-IL1β. ***, *p* < 0.001, ****, *p* < 0.0001. ns, not significant; ND, normal diet; HFD, high-fat diet.

As shown in Figure 7A, the Western blot analysis displayed that the expression levels of NLRP3 (Figure 7B), GSDMD-NT (Figure 7D), and mature-IL1β (Figure 7F) exhibited notably elevated levels compared to the ND group, and the expression level of GSDMD-FL (Figure 7C) was notably reduced than that in the ND group, suggesting that the pyroptosis occurred in the skeletal muscle fibers of SOB zebrafish. These results indicated that inflammasome-mediated pyroptosis may be involved in the development of SOB.

## 4. Discussion

Sarcopenic obesity (SOB) has been an increasing health problem worldwide. The pathogenesis of sarcopenia is complicated, involving insulin resistance, inflammation, hormonal changes, and mitochondrial dysfunction [38]. Investigating the molecular mechanisms of sarcopenic obesity is critical to advancing mechanistic research and clinical trials that aim to elucidate its pathophysiology and develop targeted interventions. In this study, we fed zebrafish with a high-fat diet to establish sarcopenic obesity. After a 16 16-week trial, HFD zebrafish displayed an obese phenotype with fat accumulation in skeletal muscle and decreased swimming capacity. In addition, the results showed that the mechanisms of sarcopenic obesity were related to impaired mitophagy and the induction of pyroptosis.

Due to its resemblance in structure and function (myofiber and sarcomere ultrastructural levels and contractile properties) to humans, zebrafish have been utilized as a model organism to study various diseases, including obesity [39]. In this experiment, we showed that zebrafish fed with a high-fat diet exhibited a remarkable increase in body weight, fat distribution, and lipid accumulation (TG and TC). Abnormal circulating TC levels are associated with muscle weakness in patients with cirrhosis [40]. We further confirmed that skeletal muscle atrophy (fast muscle fibers) and decreased swimming capacity occurred in HFD zebrafish. These data suggest that 16 weeks of an HFD could induce sarcopenic obesity in zebrafish.

As the critical organelles in myocytes, mitochondria are responsible for regulating the metabolic status of skeletal muscle [41]. Mitochondria are characterized by significant plasticity by adapting their volume, structure, and function when suffering from disuse, aging, and diseases [42,43]. For instance, in Obesity-Associated Cardiomyopathy, lipid overload induced abnormal mitochondrial morphology and decreased fatty acid β-oxidation and respiratory capacity [44,45]. Current studies also demonstrated that the accumulation of intramuscular lipids is related to the impaired skeletal muscle mitochondrial content and function [46]. Impaired mitochondrial function may lead to reduced fatty acid oxidation and oxidative phosphorylation, resulting in fat accumulation [47]. In this study, we used TEM to examine skeletal muscle mitochondrial morphology in high-fat diet-induced obese zebrafish and found that SOB zebrafish skeletal muscle mitochondria displayed severe swelling and cristae loss compared to the ND group. And, mitochondrial respiration instrument tests revealed that the Complex I and II activities were strongly inhibited in SOB zebrafish skeletal muscle. These results further confirm that the impairment of mitochondrial content and function is associated with the pathological development of SOB [47].

As an autophagic response, mitophagy specifically targets and degrades damaged mitochondria [24]. Owing to the vital role of mitochondria in the bioenergetics of the skeletal muscle system, normal operation of mitophagy is particularly important for skeletal muscle homeostasis in health and disease [33,48,49]. Obesity could result in defective mitophagy and impaired mitochondrial quality control in white adipose tissue [50] and the liver [51]. In line with this viewpoint, defective mitophagy has been shown to exacerbate the development of skeletal muscle disorders [52,53]. As the initiation of the mechanism of autophagy, the PTEN-induced putative kinase 1 (PINK1)/Parkin (PRKN) pathway primarily regulates ubiquitin-dependent mitophagy and is essential for many aspects of mitochondrial physiology [54]. Mechanically, PINK1 accumulates on the surface of damaged mitochondria while recruiting and activating the E3 ubiquitin ligase activity of PRKN and finally initiating mitophagy [55,56]. In our study, we detected the downregulation of PINK1 and PRKN in SOB zebrafish skeletal muscle. These data were consistent with the findings of impaired mitophagy in other diseases [57,58,59]. These results indicated that the regulation of mitophagy may become a new direction for the treatment of SOB.

Pyroptosis is an inflammatory form of cell death that is triggered by gasdermin (GSDM) family proteins, specifically GSDMD and GSDME. This phenomenon is marked by the activation of the NOD-like receptor protein 3 (NLRP3) inflammasome, along with the creation of cell membrane pores and the secretion of IL-1β and IL-18 [60,61]. At the cellular level, pyroptosis is characterized by rapid plasma membrane rupture and the release of pro-inflammatory intracellular contents. Studies have indicated the critical role of pyroptosis in regulating many inflammatory disorders, such as atherosclerosis, diabetes, metabolic syndrome, and nonalcoholic steatohepatitis (NASH) [61,62,63]. Recent findings highlight that obesity is often accompanied by a chronic inflammatory response [64,65], and researchers are starting to focus on pyroptosis in the pathogenesis of obesity [66]. Increased muscular pyroptosis also contributes to the pathology of acute and chronic muscle diseases [67]. In our study, TEM exhibited that mitochondria were severely swollen and had cristae loss, which were the hallmark ultrastructural features of pyroptosis. We detected the upregulation of NLRP3, GSDMD-NT, and mature-IL1β in SOB zebrafish skeletal muscle. These results confirmed that pyroptosis was triggered by the NLRP3/GSDMD signaling pathway in SOB. Previous studies highlight the roles and significance of mitochondria in pyroptosis to provide unexplored strategies. Specifically, studies have unveiled that damaged mitochondria accumulation induced by impaired mitophagy contributes to the excessive ROS and the release of oxidized mtDNA, which reportedly activates NLRP3 inflammasome and induces pyroptosis [22,68,69,70]. In our study, we found that impaired mitophagy was accompanied by pyroptosis in SOB zebrafish skeletal muscle. These suggest that impaired mitophagy may trigger pyroptosis and further promote the development of SOB.

## 5. Conclusions

In summary, our study fed zebrafish with 16 weeks of a high-fat diet to establish SOB. Our study revealed that impaired mitophagy and pyroptosis were associated with the pathogenesis of SOB. Since gender may influence the obese and sarcopenic phenotypes, this experiment did not explore the influence of gender on sarcopenic obesity, and further investigation is needed. Therapeutic strategies targeting mitophagy-mediated pyroptosis may prove useful for SOB.

## Data Availability

The data presented in this study are available on request from the corresponding author due to lab rules and regulations.
